# *Bacillus cereus* Enhanced Medicinal Ingredient Biosynthesis in *Glycyrrhiza uralensis* Fisch. Under Different Conditions Based on the Transcriptome and Polymerase Chain Reaction Analysis

**DOI:** 10.3389/fpls.2022.858000

**Published:** 2022-06-02

**Authors:** Yu Zhang, Duoyong Lang, Wenjin Zhang, Xinhui Zhang

**Affiliations:** ^1^College of Pharmacy, Ningxia Medical University, Yinchuan, China; ^2^Shaanxi Academy of Traditional Chinese Medicine, Shaanxi Traditional Chinese Medicine Hospital, Xi’an, China; ^3^Laboratory Animal Center, Ningxia Medical University, Yinchuan, China; ^4^Ningxia Engineering and Technology Research Center of Regional Characterizistic Traditional Chinese Medicine, Ningxia Collaborative Innovation Center of Regional Characterizistic Traditional Chinese Medicine, Key Laboratory of Ningxia Minority Medicine Modernization, Ministry of Education, Yinchuan, China

**Keywords:** *Bacillus cereus*, *Glycyrrhiza uralensis*, secondary metabolite pathways, transcriptome, PCR

## Abstract

The aim of this study was to evaluate the effect of *Bacillus cereus* (*B. cereus*) on the seedling growth and accumulation of medicinal ingredients of *Glycyrrhiza uralensis* Fisch. (*G. uralensis*) under control and salt stress conditions. Our results revealed the different effects of *B. cereus* on the seedling growth and accumulation of medicinal ingredients particularly in different conditions based on the transcriptome and polymerase chain reaction (PCR) analysis. Under the control condition, *B. cereus* significantly increased the expression level of the β*-AS*, *SQS*, *CHS*, *LUS*, *UGAT*, *CYP72A154*, and *CYP88D6* genes and liquiritigenin content. Under salt stress, *B. cereus* significantly increased root length and lateral root number of *G. uralensis* seedlings, the expression level of *HMGR*, β*-AS*, *CHS*, *LUS*, *UGAT*, *CYP72A154*, *CYP88D6*, and *SE* genes, and the contents of glycyrrhizic acid and glycyrrhetinic acid. Notably, the effect of *B. cereus* on the seedling growth and the medicinal ingredient biosynthesis was different under control and salt stress conditions. Specifically, the effect of *B. cereus* on the seedling growth under salt stress was greater than that under the control condition. Moreover, *B. cereus* increased liquiritigenin content under the control condition, which is closely related to flavone and flavonol biosynthesis, while it increased the contents of glycyrrhizic acid and glycyrrhetinic acid under salt stress, which is closely related to phenylpropanoid biosynthesis, and the MVA pathway is also involved. All in all, endophytes *B. cereus* could be used as a sustainable tool to develop effective bioinoculants to enhance the contents of medicinal ingredients in *G. uralensis*.

## Introduction

*Glycyrrhiza uralensis* Fisch. (*G. uralensis*), used as a common Chinese medicinal herb, contains abundant pharmacological active substances, which have attracted more attention (Hosseinzadeh and Nassiri-Asl, 2016). In the desert, although often suffering from drought and salt stresses, it is still an important sand fixation and windbreak plant (Zhou et al., 2020). In recent years, the wild resources are sharply declining and turning endangered, so the market demand has been markedly increasing for the cultivated *G. uralensis* (Chen et al., 2017). Therefore, improving the quality, yield, and stress tolerance of *G. uralensis* cultivated is the key task.

The glycyrrhizic acid and liquiritin biosynthesis pathway of *G. uralensis* is well known. In this pathway, HMGR catalyzes HMG-CoA and NADPH into MVA first, and farnesyl diphosphate (FDP), squalene synthase enzyme (SQS), and squalene epoxidase (SE) were involved in the subsequent reaction (Yan et al., 2014; Zheng et al., 2014). Then, β-amyrin synthase (β-AS), lupeol synthase (LUS), and cycloartenol synthase (CAS) are responsible for the synthesis of triterpene saponins, betulinic acid, and phytosterols, respectively (Mochida et al., 2017). Specifically, cytochromes P450s, UGAT, and CHS play crucial roles in the final step of major medicinal ingredient biosynthesis in *G. uralensis* (Xu et al., 2016; Chen et al., 2017). The content of compounds and the expression level of key genes have significantly changed in glycyrrhizic acid and liquiritin biosynthesis pathway during abiotic stress, salt stress specifically caught our attention (Wang et al., 2015, 2017; Zhou et al., 2017; Yeo et al., 2018; Kaur and Ganjewala, 2019). Shirazi et al. (2019) reported that salt stress significantly upregulated the expression level of *SQS*, β*-AS*, *CAS*, *LUS*, and some *CYPs* genes and increased the content of glycyrrhizin in *Glycyrrhiza glabra*, which illustrated that salt stress inhibited in the glycyrrhizin biosynthesis pathway. In conclusion, we provided a basis for further studies concerned with the relation between salt stress and glycyrrhizic acid pathway and liquiritin pathway in *G. uralensis*.

Endophytes colonized the internal tissues of host plants, promoted growth conditions, improved stress tolerance, and so on, so it is regarded as a low-cost and eco-friendly technology to enhance crop productivity under salt stress (Vaishnav et al., 2019). In promoting the growth of plant aspect, *Mesorhizobium* sp. and *Pseudomonas extremorientalis* endophytes could improve yield, nodule numbers, and nitrogen contents of shoot and root in salt-stressed *G. uralensis* (Egamberdieva et al., 2016). In promoting compound biosynthesis of plant aspect, endophyte *Methylobacterium oryzae* regulated ethylene metabolism in salt-stressed *Oryza sativa* by improving H^+^ ATPase activity and decreasing 1-Aminocylopropane-1-carboxylic acid (ACC) accumulation and ACC oxidase activity (Chatterjee et al., 2019). Also, endophytes increased withanolide A content in *Withania somnifera*, and significantly upregulated the expression level of genes in withanolide and sterol biosynthetic pathways including *HMGR*, *DXR*, *FPPS*, *SQS*, *SQE*, *CAS*, *SMT1*, *STE1*, and *CYP710A1* (Kushwaha et al., 2019). In conclusion, endophytes can effectively mitigate high-salinity stress and improve the stress tolerance of plants, but the effect of *Bacillus cereus* on *G. uralensis* is still not fully reported, particularly in the glycyrrhizic acid and liquiritin biosynthesis pathway aspect.

Consequently, in this study, we hypothesized that *B. cereus* can promote the growth of seedlings and increase the contents of medicinal ingredients (liquiritin, liquiritigenin, isoliquiritigenin, glycyrrhizic acid, and glycyrrhetinic acid) of *G. uralensis* under control and salt stress conditions. Furthermore, we want to explain the effect that *B. cereus* increased the contents of medicinal ingredients under different conditions based on the molecular level. Finally, we expected to unravel the underlying effect for the interactions of *B. cereus* and salt stress, provide an efficient solution for promoting the quality of *G. uralensis* cultivation, and set a solid foundation for *B. cereus* promoting the biosynthesis of glycyrrhizic acid and liquiritin.

## Materials and Methods

### Preparation of *Bacillus cereus*

The obtained target strain was isolated and selected from the early stage *G. uralensis* and identified by partial sequencing of 16SrDNA as *B. cereus* by Bioengineering (Shanghai) Co., Ltd. This strain was deposited at the China General Microbiological Culture Collection Center (No. CGMCC No. 16671). The strain was stored in 25% glycerol solution at −70°C until use. The bacterial culture suspension was incubated at 28°C for 2 days in 180 rpm, on a shaking incubator until bacteria density reached 10^8^ cfu ml^–1^.

### Plant Growth Condition and Treatment

*Glycyrrhiza uralensis* seeds were obtained from Ningxia Academy of Agriculture and Forestry Sciences, China, in September 2018. The seeds were steeped with 85% H_2_SO_4_ for 2.5 h, then 0.1% H_2_O_2_ surface-sterilized for 15 min, distilled water rinsed three times, and distilled water imbibed for 8 h at room temperature. Then, 50 seeds were sown in a 500 ml flowerpot (contains 400 g of sand) not containing any nutrients and applied to different treatment fluids separately. Then, pre-irrigated with distilled water (300 ml) as the control group and distilled water containing 75 mM NaCl as the salt stress group. Germination experiments were carried out in an incubator maintained at 28°C/20°C (day/night) with a 12 h photoperiod at a light intensity of 37.5 μmol m^–2^ s^–1^ PAR. After 23 days, *B. cereus* treatment was initiated. The bacteria culture suspension with *B. cereus* was centrifuged for 15 min at 8,000 rpm and washed with sterile distilled water, and then the optical density of the *B. cereus* at 600 nm was adjusted to 1 (∼10^8^ cfu ml^–1^). Then, both CK and S groups were divided into two subgroups and were watered with either 300 ml distilled water or 300 ml of distilled water containing *B. cereus* (10^8^-cfu ml^–1^). Six replications of all treatments were used.

At 7 days after *B. cereus* treatment, all the seedlings were collected from the flowerpot. Then, growth indicators were recorded, some were oven-dried at 60°C for 48 h and weighed, and some samples were used for HPLC analysis. Some seedlings were taken out and soil particles adhering to the root surface were gently removed with distilled water, for monitoring the colonization of *B. cereus*. Additionally, some samples were immediately stored at −80°C for transcriptomic and real-time PCR.

### Observation of Colonization on *Bacillus cereus*

Samples of root segments (1 cm) were fixed in a mixture of 3% (v/v) glutaraldehyde and 2% (w/v) paraformaldehyde in 0.1 M phosphate buffer (pH = 7), overnight at room temperature. Samples were subsequently postfixed with 1% (w/v) osmium tetroxide in the same buffer for 1 h at 4°C and dehydrated in a graded ethanol series before being embedded in SPURR resin. Ultrathin sections were contrasted with uranyl acetate and lead citrate and examined with a Hitachi H-7650 TEM at 80 kV (Vigani et al., 2010).

### Measurement of Content on Major Active Compounds

The standard of all active compounds was purchased from Shanghai Jianglai Biological Technology Co., Ltd. Preceding HPLC analysis, extracts were dissolved in a small volume of 70% ethyl alcohol and filtered using a 0.22 μm microporous membrane. A 20 μl aliquot of each sample extract was analyzed *via* HPLC (Agilent 1260 Infinity II) at 25°C. The determination of liquiritin, liquiritigenin, isoliquiritigenin, glycyrrhizic acid, and glycyrrhetinic acid was performed according to the Zheng et al. (2013) method. Acetonitrile–0.1% aqueous solution of phosphoric acid was used as the mobile phase for gradient elution, the eluent was collected with the gradient-wavelength assay, and the flow rate was 0.6 ml min^–1^, followed by measurement of UV absorbance at 254 nm. Analysis of variance was performed on the data using SPSS software (version 18.0), and significant differences compared with the control values were determined using the Duncan’s multiple range test.

### Transcriptome Sequencing

Of note, 1 μg total RNA was prepared for cDNA libraries using the protocol provided by Oxford Nanopore Technologies (ONT). In brief, the SuperScript IV First-Strand Synthesis System (Invitrogen) was used for full-length mRNA reverse transcription and following cDNA PCR for 14 circles with LongAmp Tag (NEB). The PCR products were then subjected to FFPE DNA repair and end-repair (NEB) steps and following adaptor ligation using T4 DNA ligase (NEB). Agencourt XP beads were used for DNA purification according to the ONT protocol. The final cDNA libraries were added to FLO-MIN109 flow cells and were run on the PromethION platform at Biomarker Technology Company (Beijing, China).

### Transcriptome Assembly and Annotation

Raw reads of fastq format were first processed through in-house Perl scripts. In this step, clean reads were obtained by removing reads containing adapter, reads containing ploy-N, and low-quality reads from raw data. At the same time, Q30 and sequence duplication levels of the clean data were calculated. All the downstream analyses were based on clean data with high quality. The adaptor sequences and low-quality sequence reads were removed from the data sets. Raw sequences were transformed into clean reads after data processing. These clean reads were then mapped to the reference genome sequence. Only reads with a perfect match or one mismatch were further analyzed and annotated based on the reference genome. Hisat2 tools soft were used to map with the reference genome. Gene function was annotated based on the following databases: all the plant proteins in the NCBI NR database in performing the homology search, and for each sequence, we selected the closest match for analysis. We used KOBAS software to test the statistical enrichment of differential expression genes in KEGG pathways (Kanehisa et al., 2004).

### Differential Expression Analysis

The threshold of *p*-value < 0.05 and log2 (fold change)| > 1.5 were set as the two criteria for significantly differential expression.

### Real-Time PCR

The total RNA was extracted using the RNAprep Pure Plant Kit (TIANGEN, Beijing, China) from *G. uralensis* seedlings in each experimental group. Then, 200 μg of total RNA from each sample was reverse transcribed to single-stranded cDNA using the FastQuant RT Kit (TIANGEN) according to the manufacturer’s protocol. The primers were designed using the Primer Premier 5 software (premier biosoft, Palo Alto, CA, United States; Table 1). For RT-PCR analysis, the target genes were amplified using the PCR amplification system (ABI 2720, Carlsbad, CA, United States) under the following conditions: 95°C for 4 min followed by 30 cycles consisting of 95°C for 20 s, 58°C for 20 s, 72°C for 20 s, and, 72°C for 10 min. The PCR products were analyzed using the FR-980B gel imaging analysis system (FuRi Science & Technology Co., Ltd., Shanghai, China) after 1% agarose gel electrophoresis. *β-Actin* gene was used as the reference gene.

**TABLE 1:** Oligonucleotide sequences.

Name	GenBank accession number	Sequence (5′ to 3′)
β-actin	EU190972	F: GAATTGCGTGTTGCTCCTG
		R:TGTACGACCACTGGCATAAAGA
SQS1	GQ266154.1	F: GGTCACTAATGCTTTGTTGC
		R: TAACTACACCTCCGAAGACT
β-AS	AB037203.1	F: ACAGAGAGAGGATGGTGGAT
		R: GCCAATCACCCTCTTCCAAT
HMGR	GQ845405.1	F: TGGTGCTCTTGGTGGGTTC
		R: AGGTCCTTGCCATCATTCACT
CHS	EF026979.1	F:GAGAACAACAAAGGTGCTCGTG
		R:GTACTGGGTCAGAACCAACAATG
LUS	AB663343.1	F:TCCCTGATTACTTCTGGCTCG
		R:CTTTAGTCCTTCTGCGGTGC
UGAT	KT759000.1	F:GCAGATGAGCGACTTCCTTG
		R:CCCGTGGTTTTCTCGTAGTGT
CYP72A154	AB558153.1	F:CTCAGTTGTTGGCAAAGAAAGG
(CYP5)		R: GGTGTTGGTGGGTGAAGTCTAA
CYP88D6	AB433179.1	F:TTTACATTCCACAAAGCACTCG
(CYP6)		R: CGCTAATATCCTCGTCCTCCA
SE	MG763680.1	F:CTTGGTCCTCCACTTCTTCG
		R:ATTTGTCTCACTCCTTCAGCCT

### Statistical Analysis

All experimental data were analyzed using ANOVA, SPSS 17.0 software (SPSS Inc., Chicago, IL, United States). Significant differences were tested using the least significant difference (LSD) test at *p* < 0.05. Mean values and standard errors (SEs) were presented. Pearson’s correlation analysis was performed when the variables were approximately normally distributed and had no outliers.

## Results

### Effect of *Bacillus cereus* on the Growth Condition of *Glycyrrhiza uralensis* Seedlings

**FIGURE 1:**
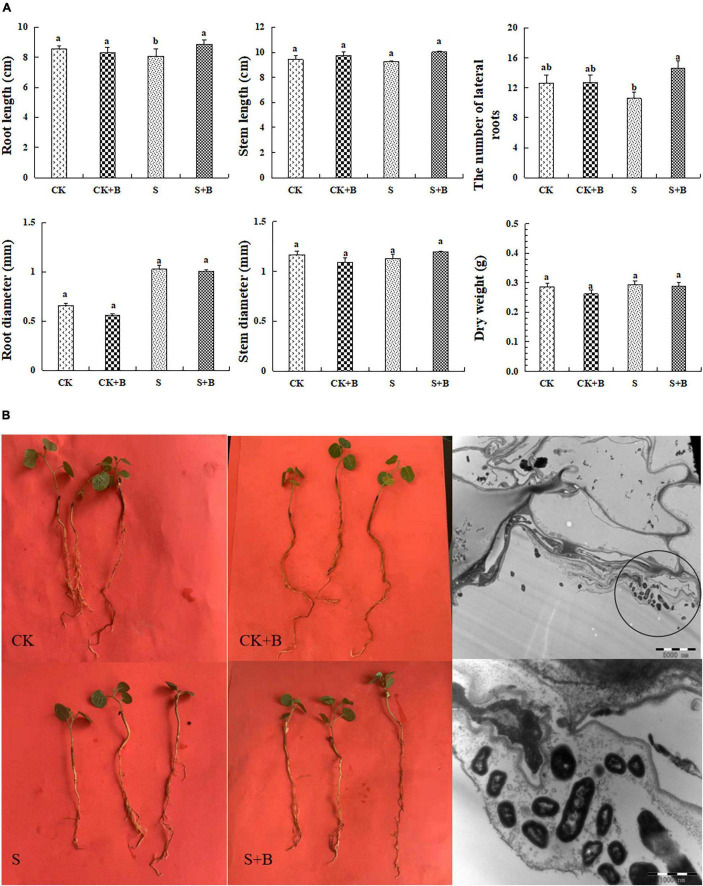
**(A)** Effect of *B. cereus* on seedlings growth of *G. uralensis* under control (CK) and salt stress (S) conditions. Data are expressed as the mean ± SE (*n* = 6). The different letters within the different treatments indicate the significant difference at *P* < 0.05. **(B)** The growth condition of seedlings under different treatments and colonization of *B. cereus* in the roots in *G. uralensis.*

The growth condition of *G. uralensis* seedlings and colonization of *B. cereus* are shown in Figures 1A,B. Salt stress significantly inhibited the root length of *G. uralensis* seedlings. The effect of *B. cereus* on seedling growth under salt stress was greater than that under the control condition. Under salt stress, *B. cereus* significantly increased the root length and the number of lateral roots (Figure 1A). In conclusion, *B. cereus* significantly improved the growth condition of *G. uralensis* seedlings under salt stress.

### Effect of *Bacillus cereus* on the Content of Major Active Compounds

**FIGURE 2:**
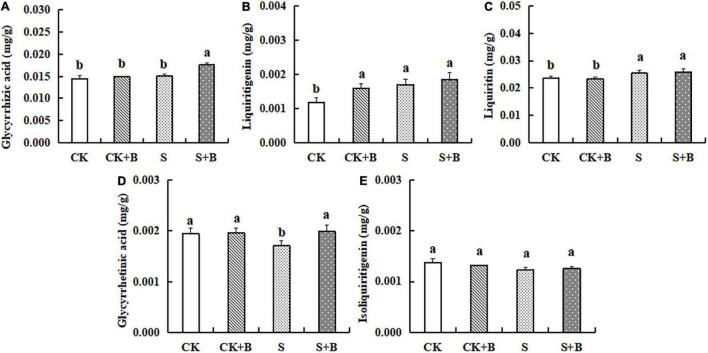
Effect of *B. cereus* on the content of **(A)** glycyrrhizic acid, **(B)** liquiritigenin, **(C**) liquiritin, **(D)** glycyrrhetinic acid, and **(E)** isoliquiritigenin of *G. uralensis* under control (CK) and salt stress (S) conditions. Data are expressed as the mean ± SE (*n* = 6). The different letters within the different treatments indicate the significant difference at *P* < 0.05.

As shown in Figure 2, compared with the control treatment, salt stress significantly increased the content of liquiritin and liquiritigenin. Compared with the salt stress, the content of glycyrrhetinic acid showed a higher level under the control treatment. The effect of *B. cereus* on the content of major active compounds under salt stress was greater than those under control conditions. Under the control condition, *B. cereus* significantly increased liquiritigenin content. Under salt stress, *B. cereus* significantly increased the contents of glycyrrhizic acid and glycyrrhetinic acid.

### Transcriptomic Data Analysis for the Effect of *Bacillus cereus* on *Glycyrrhiza uralensis*

**FIGURE 3:**
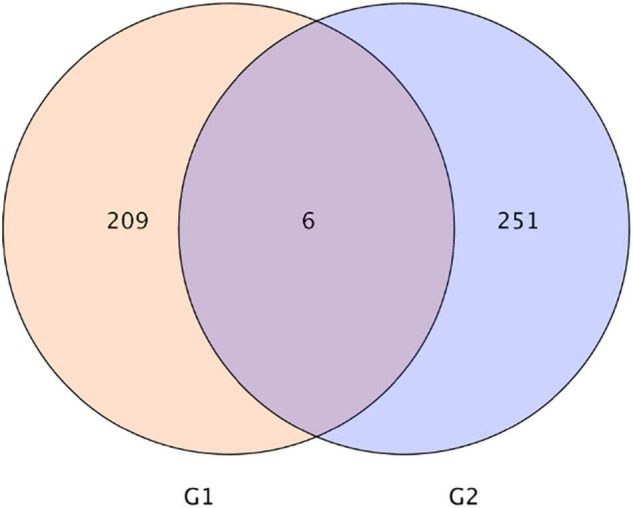
|Venn diagram of DEGs of control condition combined with *B. cereus* (CK+B) vs control (CK) and salt stress combined with B. cereus (S+B) vs salt stress (S) comparison in *G. uralensis*. G1, DEGs of CK+B vs CK; G2, DEGs of S+B vs S.

**FIGURE 4:**
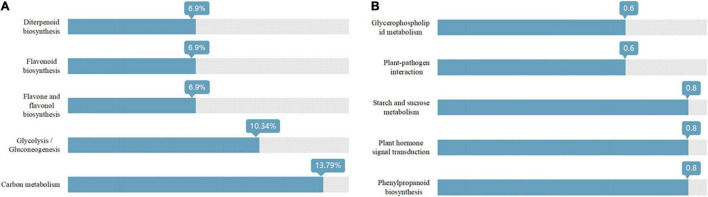
KEGG annotation of the DEGs (only up-regulated) in control condition combined with *B. cereus* (CK+B) vs control (CK) **(A)** and salt stress combined with B. cereus (S+B) vs salt stress (S) **(B)** comparisons in G. uralensis (Selected the top five items).

As shown in Figure 3, the Venn diagram of the expression level of upregulated gene comparisons was found by caused *B. cereus* under control and salt stress conditions. *B. cereus* upregulated the expression level of 215 genes under the control condition and upregulated the expression level of 257 genes under salt stress. In the KEGG database, the upregulated DEGs by *B. cereus* under the control condition were mainly enriched in carbon metabolism (13.79%), glycolysis/gluconeogenesis (10.34%), flavone and flavonol biosynthesis (6.9%), flavonoid biosynthesis (6.9%), and diterpenoid biosynthesis (6.9%; Figure 4A). Additionally, the upregulated DEGs by *B. cereus* under salt stress were mainly enriched in phenylpropanoid biosynthesis (8.0%), plant hormone signal transduction (8.0%), starch and sucrose metabolism (8.0%), plant–pathogen interaction (6.0%), and glycerophospholipid metabolism (6.0%; Figure 4B).

### Effect of *Bacillus cereus* on Gene Expression of Glycyrrhizic Acid and Liquiritin Biosynthesis Pathway

**FIGURE 5:**
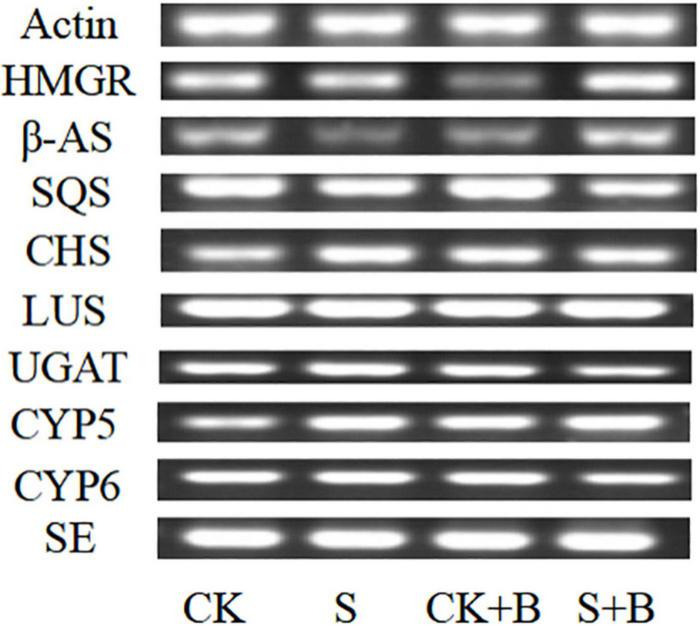
Expression level of glycyrrhizic acid and liquiritin biosynthesis pathway-related genes in *G. uralensis* seedlings under control (CK), salt stress (S), control condition combined with *B. cereus* (CK+B), and salt stress combined with *B. cereus* (S+B).

As shown in Figure 5, compared with the control condition, the expression level of *HMGR*, β*-AS*, *SQS*, and *SE* genes was lower level, but CHS and CYP5 were higher under salt stress. *B. cereus* enhanced the gene expression level of glycyrrhizic acid and liquiritin biosynthesis pathway, but the effect of *B. cereus* was different under control and salt stress conditions. The expression level of *SQS*, *CHS*, and *CYP5* genes was higher caused by *B. cereus* under the control condition compared with the control, and the expression level of *HMGR*, β*-AS*, and *CYP5* genes was higher under salt stress compared with the salt stress.

**FIGURE 6:**
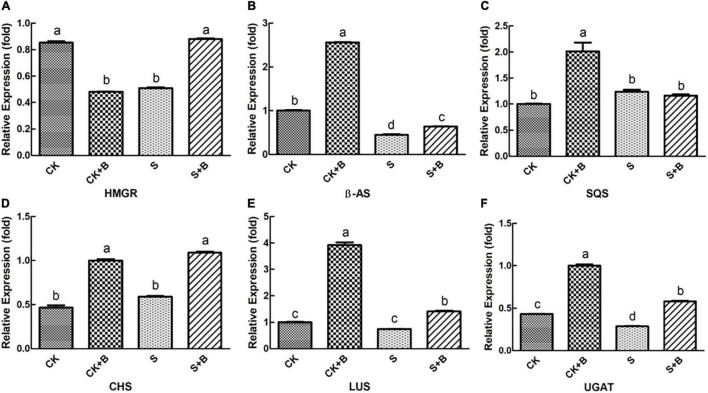
Effect of *B. cereus* on expression level of **(A)** HMGR, **(B)** β-AS, **(C)** SQS, **(D)** CHS, **(E)** LUS, and **(F)** UGAT genes of *G. uralensis* under control (CK) and salt stress (S) conditions. Data are expressed as the mean ± SE (*n* = 6). The different letters within the different treatments indicate the significant difference at *P* < 0.05.

**FIGURE 7:**
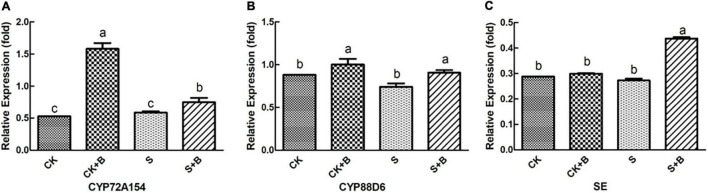
Effect of *B. cereus* on expression level of **(A)** CYP72A154, **(B)** CYP88D6, and **(C)** SE genes of *G. uralensis* under control (CK) and salt stress (S) conditions. Data are expressed as the mean ± SE (*n* = 6). The different letters within the different treatments indicate the significant difference at *P* < 0.05.

As shown in Figures 6, 7, compared with the control condition, the highest fold-decrement in the β*-AS* was observed under salt stress (0.55-fold decrease), followed by *HMGR* (0.34-fold decrease) and *UGAT* (0.14-fold decrease). While the highest fold-increment in the *LUS* was observed (2.92-fold increase) under control condition inoculation with *B. cereus*, followed by β*-AS* (1.56-fold increase), *SQS* (1.01-fold increase), *UGAT* (0.57-fold increase), *CYP72A154* (0.55-fold increase), *CHS* (0.53-fold increase), and *CYP88D6* (0.12-fold increase). In contrast, the highest fold increment in the *LUS* was observed (0.67-fold increase) under salt stress inoculation with *B. cereus*, followed by *CHS* (0.50-fold increase), *HMGR* (0.37-fold increase), *UGAT* (0.29-fold increase), β*-AS* (0.19-fold increase), *CYP88D6* (0.17-fold increase), *SE* (0.17-fold increase), and *CYP72A154* (0.16-fold increase).

### Correlation Analysis and Principal Component Analysis

**FIGURE 8:**
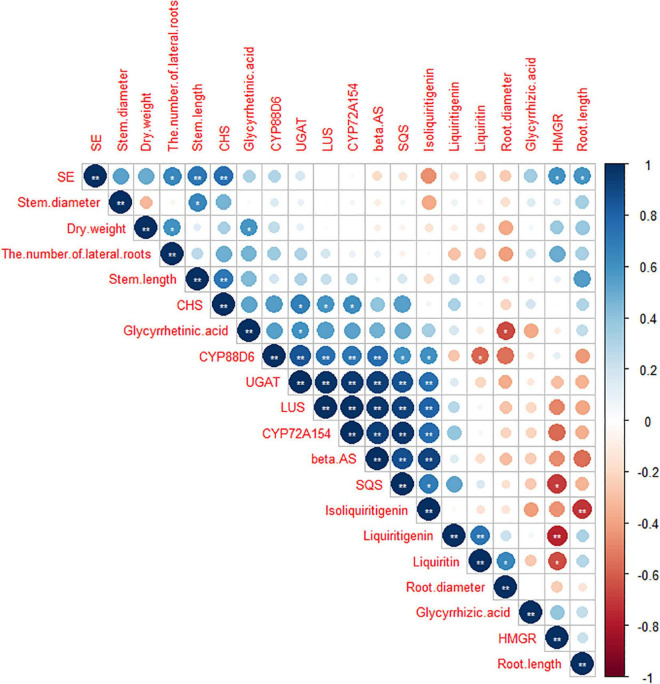
Correlation analysis among seedlings growth, medicinal ingredients, and secondary metabolites pathway in *G. uralensis* under four treatments. **Correlation is significant at the 0.01 level (two-tailed). *Correlation is significant at the 0.05 level (two-tailed).

**FIGURE 9:**
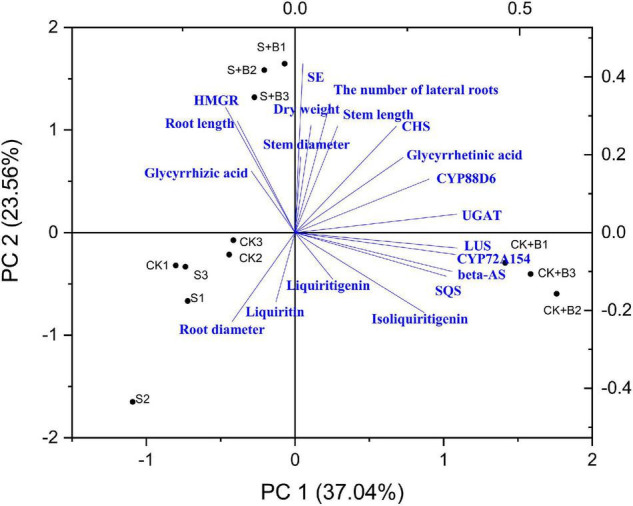
Principal component analysis (PCA).

As shown in Figures 7, 8, data of groups of very significant positive relations (*p* < 0.01) were observed. Additionally, data of 3 groups of significant positive relations (*p* < 0.05) were observed. In Figure 9, the first and second principal components (PCs), respectively, reflect 37.04 and 23.56% variation of the experimental data and altogether explain 60.60% variation. The biosynthesis pathway of glycyrrhizic acid majorly contributes to PC2 and PC1 formation. The sample distribution within the PC1/PC2 plane is heterogeneous, and PC2 clearly separated *B. cereus*-treated plants under the salt stress condition from control and salt-treated plants due to regulation of the biosynthesis pathway of glycyrrhizic acid under salt stress. PC1 significantly separated *B. cereus*-treated plants under the control condition from control and salt-treated according to the similar effect.

## Discussion

In general, salt is a necessary nutrient element for the normal growth of plants and also plays an important role in the physiological and biochemical functions of plants (such as osmotic adjustment, antioxidant system, and carbon and nitrogen metabolism), but excessive concentration will cause plant death (Jose et al., 2017; Kanagaraj and Sathish, 2017). Salt stress inhibited the root growth of plants, reduced the activity of root tip cells mitotic, and increased abnormalities such as chromosome breaks and bridges and formation of micronuclei, which belongs to the cytotoxic effect (Kiełkowska, 2017). In this experiment, salt stress significantly decreased the root length of seedlings. Salt stress would affect the absorption of water and nutrients by damaging the roots of plants and further affect the supply of water and nutrients above the ground (Guo et al., 2015). Surprisingly, one biological method is the application of plant growth-promoting bacteria to decrease the deleterious effect of salinity (Trdan et al., 2019). Under salt stress, *B. cereus* significantly increased the root length and the number of lateral roots, but have no significant effect on dry weight, which could attribute to need more time for accumulate the effect of inoculation *B. cereus* on dry weigh. Plant growth-promoting bacteria that can alleviate salt stress have been reported in pepper (Amor and Cuadra-Crespo, 2012), barley (Suarez et al., 2015), okra (Habib et al., 2016), and *Medicago sativa* L. (Ansari et al., 2019). In addition, we also found that the effect of *B. cereus* on regulating the growth condition was greater under salt stress than these under the control condition, probably because the microorganisms can secrete ACC deaminase, degrade ethylene synthesis precursors, and regulate ethylene concentration under salt stress, which is an important mechanism for microorganisms to enhance plant stress resistance (Pande et al., 2016). In summary, *B. cereus* can promote the growth of *G. uralensis* seedlings, but the effect was greater under salt stress than that under the control condition.

In the past, Ponce et al. (2004) observed inoculation of *Glomus intraradices* increased flavonoid content from roots and shoots of *Trifolium repens* through increasing the root colonization, which may refer to the plant metabolism to secrete different flavone derivatives. At present, it is difficult to explain meticulously the molecular mechanism of endophytes on active compound biosynthesis because it is difficult to evaluate the potential functional relationships in traditional Chinese medicine, so we will try to analyze the molecular effect of *B. cereus* on improving the content of active compounds in *G. uralensis* based on transcriptome and PCR results. First, we observed that *B. cereus* increased glycyrrhizin content under the control condition and the contents of glycyrrhizic acid and glycyrrhetinic acid under salt stress (Figure 2). Second, our KEGG annotation results revealed that *B. cereus* increased liquiritigenin content is closely related to regulate flavone and flavonol biosynthesis under the control condition (Figure 4A; Berim and Gang, 2016), and *B. cereus* increased contents of glycyrrhizic acid and glycyrrhetinic acid are closely related to the regulation of phenylpropanoid biosynthesis under salt stress (Figure 4B; Li et al., 2016).

Liquiritigenin is one of the major active metabolites in *G. uralensis*, which belongs to flavonoid compounds and plays important physiological roles in protecting plants from biotic and abiotic stresses (Hosseinzadeh and Nassiri-Asl, 2016). Since liquiritigenin belongs to flavonoid compounds, our results explained that *B. cereus* increased the liquiritigenin content by upregulating the expression level of genes on the flavone and flavonol biosynthesis pathway under the control condition. Under the salt stress condition, *B. cereus* increased the contents of glycyrrhizic acid and glycyrrhetinic acid, this is a complicated process. These results are also consistent with our correlation analysis and PC analysis results. According to previous studies, glycyrrhizic acid and glycyrrhetinic acid are synthesized mainly by the MVA pathway, and this pathway is closely related to the cytochrome P450s (Pandian et al., 2020). Cytochrome P450s may account for the biosynthesis of triterpene saponins, phenylpropanoid, and fatty acids in plant-specific organs and further modified oxidation and glycosylation (Yendo et al., 2010; Liao et al., 2020). Phenylpropanoid compounds play a pivotal role particularly in signaling molecules both within the plant and in communications with other microbes, response to environmental conditions, and improving crop yield and quality (Ali and McNear, 2014). Also, we found that more than sixteen cytochrome P450s have been implicated in the phenylpropanoid metabolism pathway because most are derived from phenylalanine by the phenylpropanoid pathway leading to 4-coumaroyl-CoA (Pandian et al., 2020). In our study, *B. cereus* increased the contents of glycyrrhizic acid and glycyrrhetinic acid, upregulated the expression level of *CYP88D6* and *CYP72A154* genes, and upregulated the level of DEGs in phenylpropanoid biosynthesis under salt stress, which showed that glycyrrhizic acid and glycyrrhetinic acid, CYPs, and phenylpropanoid biosynthesis are closely related. Moreover, the complex relationships between *B. cereus* and glycyrrhizic acid and liquiritin biosynthesis pathway remain to be further studied.

**FIGURE 10:**
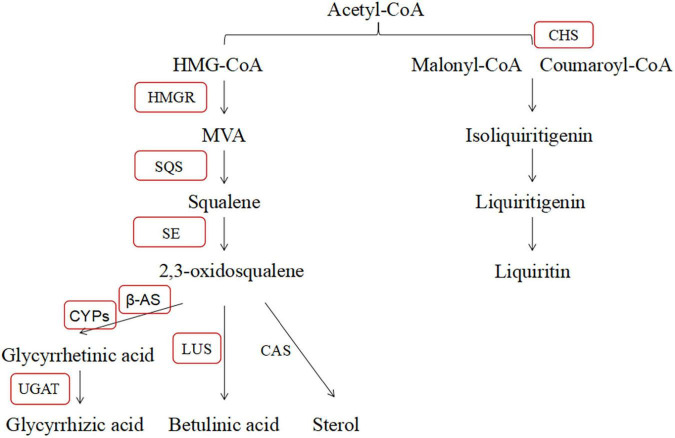
Glycyrrhizic acid and liquiritin biosynthesis pathway.

In contrast, we interpreted the effect of *B. cereus* improving the contents of active compounds of *G. uralensis* based on PCR results. According to a large number of reports, we summarized the major active compounds and the enzymes of the secondary metabolic biosynthesis pathway in *G. uralensis* (Figure 10). In past studies, the effect of high salt concentration on the content of effective ingredients depends on different plant types, growth stages, and action sites (Wang et al., 2016). Behdad et al. (2020) found that 100 mM NaCl significantly upregulated the expression level of key genes (β*-AS*, *CYP88D6*, and *CYP72A154*) in *Glycyrrhiza glabra* L. involved in triterpenoid saponin biosynthesis and directly increased the content of glycyrrhizic acid. Shirazi et al. (2019) also found that salt stress upregulated the expression level of key genes (including *SQS*, β*-AS*, *LUS*, *CAS*, *CYP88D6*, and *CYP93E6*) in Iranian licorice. Moreover, Wang et al. (2020) reported that 200 mM NaCl upregulated the expression level of key enzymes (β-AS, CYP88D6, and CYP72A154) involved in the glycyrrhizic acid biosynthesis pathway in *G. uralensis*. Our results are different from theirs, and in this study, salt stress significantly downregulated the expression level of *HMGR*, β*-AS*, and *UGAT* genes, upregulated the expression level of *CYP5* and *CHS* genes, significantly decreased glycyrrhetinic acid content, and significantly increased the contents of liquiritin and liquiritigenin, which is consistent with the biosynthesis pathway of active compounds in *G. uralensis*. Salt stress downregulated the expression level of *HMGR*, β*-AS*, and *UGAT* genes, reducing the content of compounds in glycyrrhizic acid synthesis pathway; at the same time, salt stress upregulated the expression level of *CYP5* and *CHS* genes, increasing the content of compounds involved in the liquiritin synthesis pathway.

A large number of studies have found that endophytes can improve the accumulation of active compounds in plants. Native endophytes of *Coleus forskohlii* significantly promoted the plant growth (shoot weight, oil content, and oil yield) and increased the contents of davanone and ethyl cinnamate in *Andrographis paniculata*, *Pelargonium graveolens*, and *Artemisia pallens* (Mastan et al., 2019). Endophytes *Phoma* sp., *Alternaria* sp., *Bipolaris* sp., and *Cladosporium* sp. significantly increased seed germination, promoted plant growth, and increased the contents of flavonoid and phenolic in rice (Khan et al., 2017). Particularly in the salt stress, it has been reported that endophytes have many beneficial effects on salt-stressed plants including the production of scavengers, ACC deaminase enzyme, nitrogen fixation, production of compatible solutes, antibiotics, and phytohormones (Fouda et al., 2018). Amanifar et al. (2019) found that arbuscular mycorrhizal increased the content of glycyrrhizic acid and upregulated the expression level of β*-AS* and *P450* genes in *Glycyrrhiza glabra* under salt stress. This is similar to our research results that *B. cereus* upregulated the expression level of key genes and the content of active compounds involved in the glycyrrhizic acid and liquiritin biosynthesis pathway, which indicated that *B. cereus* induced the accumulation of major active compounds in *G. uralensis* through the upregulation expression level of MVA and downstream pathway genes such as *HMGR*, β*-AS*, *SQS*, *CHS*, *LUS*, *UGAT*, *CYP72A154, CYP88D6*, and *SE*. Moreover, the regulatory effect of *B. cereus* was different under control and salt stress conditions. HMGR acts as the catalyst for the first committed reaction of the mevalonate pathway, which is critical for plant adaptations against harsh environmental conditions (Zheng et al., 2021), so the expression level of *HMGR* gene was higher by *B. cereus* under salt stress. Moreover, *B. cereus* upregulated the expression level of many key genes of *G. uralensis* in both control and salt stress conditions, but only increased the content of liquiritigenin under the control condition and increased the contents of glycyrrhizic acid and glycyrrhetinic acid under the salt stress condition. Interestingly, this result is consistent with the results of transcriptome analysis, which explained that the effect of *B. cereus* on the glycyrrhizic acid and liquiritin biosynthesis pathway of *G. uralensis* under control and salt stress conditions was different.

## Conclusion

In this study, *B. cereus* improved the growth condition and the contents of major medicinal components of *G. uralensis* seedlings. Interestingly, the effect of *B. cereus* on the seedling growth and the glycyrrhizic acid and liquiritin biosynthesis pathway was different under control and salt stress conditions. In promoting the seedling growth aspect, the effect of *B. cereus* under the salt stress condition was greater than that under the control condition. Also, the effect of *B. cereus* on the accumulation of active compounds was different under control and salt stress conditions based on transcriptome and PCR results. *B. cereus* increased liquiritigenin content under the control condition, which is closely related to regulate flavone and flavonol biosynthesis, and increased the contents of glycyrrhizic acid and glycyrrhetinic acid involved in the phenylpropanoid biosynthesis pathway under the salt stress condition. Moreover, our study provided some theoretical basis for improving the contents of major active compounds and enhancing salt tolerance of *G. uralensis* and the development and utilization of *B. cereus* and molecular breeding of *G. uralensis*, which will help the further study in *G. uralensis*.

## Data Availability Statement

The datasets presented in this study can be found in online repositories. The names of the repository/repositories and accession number(s) can be found below: https://www.ncbi.nlm.nih.gov/, GSE187003.

## Author Contributions

YZ and XZ contributed to the experiment design. WZ and YZ performed the material preparation and data collection. DL and YZ analyzed the data. YZ wrote the first draft of the manuscript. All authors commented on previous versions of the manuscript, read and approved the final manuscript.

## Conflict of Interest

The authors declare that the research was conducted in the absence of any commercial or financial relationships that could be construed as a potential conflict of interest.

## Publisher’s Note

All claims expressed in this article are solely those of the authors and do not necessarily represent those of their affiliated organizations, or those of the publisher, the editors and the reviewers. Any product that may be evaluated in this article, or claim that may be made by its manufacturer, is not guaranteed or endorsed by the publisher.
